# Redox for Repair: Cold Physical Plasmas and Nrf2 Signaling Promoting Wound Healing

**DOI:** 10.3390/antiox7100146

**Published:** 2018-10-19

**Authors:** Anke Schmidt, Sander Bekeschus

**Affiliations:** 1 Plasma Life Science, Leibniz Institute for Plasma Science and Technology (INP Greifswald), Felix-Hausdorff-Str. 2, 17489 Greifswald, Germany; 2 ZIK-PRE, Leibniz Institute for Plasma Science and Technology (INP Greifswald), Felix-Hausdorff-Str. 2, 17489 Greifswald, Germany; sander.bekeschus@inp-greifswald.de

**Keywords:** Keap1, kINPen, plasma medicine, reactive oxygen species (ROS), reactive nitrogen species (RNS), redox regulation

## Abstract

Chronic wounds and ulcers are major public health threats. Being a substantial burden for patients and health care systems alike, better understanding of wound pathophysiology and new avenues in the therapy of chronic wounds are urgently needed. Cold physical plasmas are particularly effective in promoting wound closure, irrespective of its etiology. These partially ionized gases deliver a therapeutic cocktail of reactive oxygen and nitrogen species safely at body temperature and without genotoxic side effects. This field of plasma medicine reanimates the idea of redox repair in physiological healing. This review compiles previous findings of plasma effects in wound healing. It discusses new links between plasma treatment of cells and tissues, and the perception and intracellular translation of plasma-derived reactive species via redox signaling pathways. Specifically, (i) molecular switches governing redox-mediated tissue response; (ii) the activation of the nuclear E2-related factor (Nrf2) signaling, together with antioxidative and immunomodulatory responses; and (iii) the stabilization of the scaffolding function and actin network in dermal fibroblasts are emphasized in the light of wound healing.

## 1. Introduction

Defective wound healing affects millions of people in the western world, and is a strong burden to patients and health care systems [1]. Although there is a plethora of wound therapies available [2], challenges remain in the treatment of problematic wounds [3]. Phases of wound healing are complex and dynamically regulated during physiological tissue repair; they can co-exist in different wound areas and tend to blend together depending on the degree of wound closure [4,5,6]. Moreover, several phases such as blood coagulation, thrombosis, fibrosis, migration, proliferation, and programmed cell death are dependent on reactive species-mediated signaling, redox level, and wound oxygenation [7]. Endogenous generation of oxidants leads to reversible oxidative modifications, including redox regulation, sensing, and redox signaling, which contribute to physiological or pathological stages of wound healing [8].

In comparison to normal wound healing, several studies have shown pathophysiological mechanisms counteract healing processes in chronic wounds. One parameter that has been described to aggravate wound healing is diabetes mellitus (DM), which is ranked as the 10th leading cause of death in 2012 [9], affecting 24 million people in the U.S. [10] or 382 million people worldwide (International Diabetes Federation). The incidence of diabetes continues to rise rapidly. In Germany, 3.8% of the insured population have been diagnosed with diabetic foot ulcers [11]. Extensive differences were found regarding the cellular redox level in non-healing (diabetic) wounds [8,12]. Accordingly, they display an imbalance in redox processes and are often linked to chronic inflammation [13], ultimately disturbing the sequence of events necessary for healing. For example, low-grade inflammation is involved in diabetic micro-angiopathy [14]. In type 1 diabetes, the level of IL-2 is reduced, and hence, wound healing is in a prolonged inflammatory phase [15,16]. A modulated apoptosis [17] or an increased apoptosis of T lymphocytes [18], a reduced number and activation of macrophages, which results in reduced lymphatic vessel formation [19], leads to impaired wound healing in diabetic patients [20]. In the granulation phase, DM disturbs re-epithelialization by affecting multiple proteins and genes such as angiopoietin-4 [21]. Moreover, DM has a negative influence on signaling intermediates responsible for coordination and regulation of wound healing, angiogenesis, and vasculogenesis [22], leading to a decreased expression of angiogenetic growth factors [23] and an impaired neovascularization [24]. As a result, diabetic patients are prone to the development of chronic wounds due to deficiencies in either endothelial progenitor cells or peripheral tissue homing and in engraftment of bone marrow [25]. In the matrix recovering phase, dysfunction of fibroblasts mediated by heat shock proteins (HSPs) together with aberrant collagen homeostasis contribute to impaired healing [26]. In addition, gap junctional connexins form channels between two adjacent cells and their expression is highly regulated after wound formation at transcriptional, translational, and post translational levels [27]. Upregulation of connexin proteins during diabetes leads to improper formation of gap junctions and attributes to the passage of apoptotic and inflammatory signals, thereby resulting in delayed healing of chronic diabetic ulcers [28]. In addition, dysregulation of apoptosis in response to hyperglycemia causes impaired wound healing, which can further worsen ischemia and coronary artery disease [14]. Thus, alternative therapeutic strategies are required for treatment of non-healing wounds.

Since inflammation and wound healing are subject to redox control [29], there is an obvious link to plasma medicine. By using cold physical plasmas generating or mimicking physiologically active reactive species, a beneficial strategy of tissue repair in chronic (diabetic) wounds seems possible. However, the application of cold plasma as a new medical alternative and/or supporting therapy requires the identification of the molecular switches governing redox-regulated tissue responses and key molecules as well as signaling pathways. Over the past 10 years, a huge number of in vitro studies have demonstrated plasma-triggered wound healing based on the stimulation of cell proliferation and survival [30,31], extracellular matrix (ECM) protein synthesis [32,33,34], changes of junctional proteins and cytoskeletal architecture [35], as well as apoptosis [36,37,38]. Moreover, several studies have showed that cold plasma treatment favors the redox regulation of targets known to be important in wound healing [39,40,41]. Specifically, this includes redox pathways and the regulation of redox proteins, ultimately resetting inflammation to reinitialize the sequence of events necessary for healing [42].

This article summarizes state-of-the-art of plasma medical research to understand the biological effects of plasma-generated reactive species, as well as plasma-induced signaling pathways and to further elucidate the relevance and significance of redox regulation in different stages and events of (diabetic) wound healing.

## 2. Cold Physical Plasma-Derived Reactive Species and Their Biological Effects

### 2.1. Plasma-Derived Reactive Species

Cold physical plasmas are multicomponent and complex systems that have unique features, but also differences [33,43]. Most of them are a significant source of highly reactive oxygen species (ROS) like ozone (O_3_), hydroxyl radicals (OH^−^), superoxide (O_2_^−^), and singlet oxygen (_1_O_2_), as well as nitrogen species (RNS) such as nitric oxide (NO), peroxynitrite (ONOO^−^), and nitrogen dioxide (NO_2_^−^) [44]. Some of these reactive components are being considered second messengers in the field of redox biology [45]. Additionally, unique characteristics of cold plasmas are charged particles, ions, thermal and ultraviolet radiation, as well as electrical fields [46,47]. A detailed overview about physicochemical and biological parameters plus their biological application along with a detailed risk estimation of plasma sources was recently given [48]. The most striking parameter provided by plasma in tissues is the superficial penetration depth of approximately 10 µm (especially for short-living reactive species) to 60 µm [49]. However, due to its rapid reactions with biomolecules (proteins, lipids, DNA), plasma-generated stable species have oxidizing properties, e.g., protein oxidation of redox-sensitive cysteine residues and thiol groups [50,51] evoking paracrine effects and changes of microenvironment in deeper skin layers [34]. Nevertheless, a lack of histological damage was observed after 10 min of single-spot plasma treatment of human skin biopsies [30].

**Figure 1:**
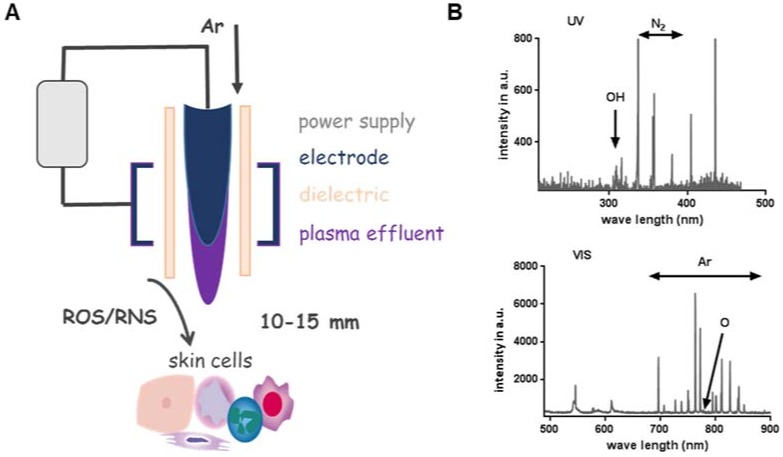

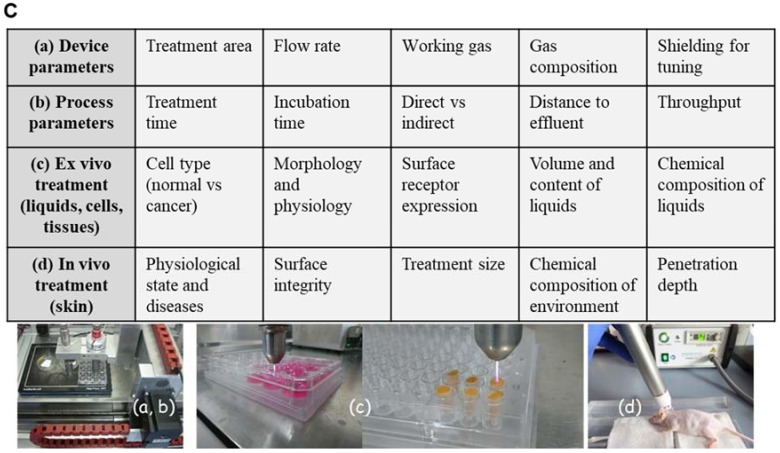
Parameter influencing impact, efficiency and specificity of plasma. (**A**) Cold physical plasma generates reactive oxygen (ROS) and nitrogen species (RNS), which can be delivered to target cells. (**B**) Optical emission spectroscopy resolves, for example, OH (~308 nm) and the second positive system of molecular nitrogen (315–380 nm, upper diagram) as well as other excited species such as atomic O (777 nm and 844 nm) and atomic Ar in the range of 696 and 912 nm (lower diagram). (**C**) Device parameters such as treatment area, flow rate, working gas, components of gas and their tuning (**a**) as well as process parameter including treatment and incubation time, direct vs. indirect treatment, distance to the effluent and throughput can be modulated. Plasma is used to treat liquids (**b**), cells or tissues (**c**) or can be applied in vivo directly to the skin (**d**).

Besides other plasma sources (e.g., dielectric barrier or microwave-based) [52], the kINPen plasma jet (neoplas tools GmbH, Greifswald, Germany) composition including power supply, electrode, dielectric and plasma effluent was depicted to show the general structure of a jet-based plasma device (Figure 1A). The pen-sized hand-held unit generates an atmospheric argon plasma primarily for spot-like treatments of target cells [53]. Plasma composition varied through the way from gaseous over liquid phase to the target [54,55]. Additionally, biological plasma effects are largely dependent on plasma-generated reactive species in the gas phase, which subsequently diffuse or react with proteins and lipids in cells or tissues. Hence, monitoring of the gas via optical emission spectroscopy phase allows monitoring of the reactive species output and biological effect. For example, biological effects of argon-oxygen-generated plasmas are mainly explained by the formation of ROS [56,57]. Based on this analytical technique, it was possible to compare spectra in the ultraviolet (UV) and visible range regarding the present plasma-excited species produced by kINPen and to standardize plasma treatment for biological applications (Figure 1B) [58]. In addition to device parameters such as treatment area, flow rate, working gas, components of gas and their tuning, process parameters can also be modulated including treatment and incubation time, direct vs. indirect treatment, distance to the target from effluents and throughput [59]. One favorable advantage of cold plasma’s use is the generation of highly active species at the site of interest (liquids, cells and tissues), where they can directly function as signaling or redox-reactive molecules (Figure 1C). In this context, the general applicability of cold plasmas to treat the skin or infected wounds has been shown in vivo [60,61,62].

### 2.2. Nrf2 Signaling in Plasma-Assisted Wound Healing

**Figure 2:**
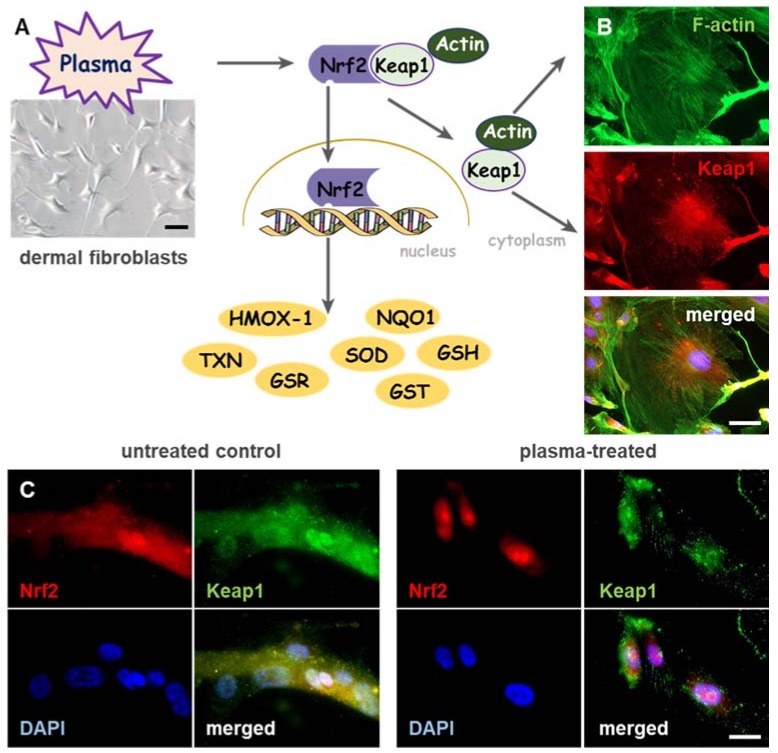
Cold physical plasma triggers nuclear translocation of Nrf2, and induces co-localization of Keap1 with actin filaments in the cytoplasm. (**A**) Dermal fibroblasts (bright field image, left) were isolated from SKH1 mouse skin and exposed to cold physical plasma-derived ROS/RNS. Upon nuclear translocation of the nuclear factor erythroid 2-related factor 2 (Nrf2), plasma significantly altered antioxidant and phase II detoxification enzymes and proteins (e.g., heme oxygenase 1 (HMOX-1), NADPH quinone oxidoreductase 1 (NQO1), thioredoxin (TXN), glutathione reductase (GSR), superoxide dismutase (SOD), glutathione *S*-transferase (GST), glutathione (GSH) etc.). (**B**) Cytoplasmic localization of Kelch-like ECH-associated protein 1 (Keap1) was detected immunohistochemically by anti-Keap1 antibody (red). Co-localization of Keap1 with actin filaments was visualized by staining with fluorescein isothiocyanate (FITC)-phalloidin (green). (**C**) Subcellular localization of Keap1 (green) and trans-localization of Nrf2 (red) from the cytoplasm to the nucleus were detected immunohistochemically by anti-Keap1 and anti-Nrf2 antibodies in plasma-treated (right panel), but not control fibroblasts (left panel). Scale bars 100 µm (**A**), 50 µm (**B**,**C**).

While plasma-induced wound healing revealed differences of a huge number of factors, selected targets were introduced. Beyond that, changes in ROS levels trigger a coordinated action of redox-sensitive transcription factors [63] underlining the importance of controlled redox signaling during wound healing. The nuclear factor erythroid 2-related factor 2 (Nrf2), a basic leucine zipper (bZIP) transcription factor, is a crucial translator for redox signaling and functions in cellular defense against imbalances in redox homeostasis. Such imbalance between the production and the detoxification of reactive intermediates affects the cellular stress level. Generally, Nrf2 activates cellular rescue pathways against oxidative injury [64,65,66], inflammation [67,68] as well as apoptosis [69,70] and plays a key role in regulation of genes, which encode detoxifying enzymes and non-enzymatic proteins [71]. Under basal conditions, Nrf2 is associated with an actin-binding protein, Kelch-like ECH-associated protein 1 (Keap1), a vital factor in Nrf2 signaling cascade, which retains Nrf2 in the cytoplasm, where it is targeted for ubiquitin-mediated degradation [72,73]. As a key regulator of oxidative and electrophilic stress, Keap1 is constitutively expressed in cytoplasmic regions of cells [74,75] as shown in dermal fibroblasts isolated from mouse skin (Figure 2A). Keap1 acts not only as an oxidative stress sensor but also functions as a regulator of F-actin filament architecture [75], supporting the assumption that Keap1 also possesses Nrf2-independent functions [76,77]. Colocalization of Keap1 (red) with actin filaments (green) promotes scaffolding functions and a controlled Nrf2 regulation in dermal fibroblasts (Figure 2B). Furthermore, plasma-mediated activation of Keap1 supports the reorganization of architecture of actin cytoskeleton and focal adhesion [35].

After release of Nrf2 from Keap1 by oxidation events at cysteine, Nrf2 translocates to the nucleus (Figure 2C), binds to specific DNA recognition sites, namely, antioxidant responsive elements (AREs) in the promoters of its target genes, and activates their transcription [78]. Most skin cells are equipped with mechanisms to detoxify reactive species via expression of antioxidant enzymes such as heme oxygenase 1 (HMOX-1), NADPH quinone oxidoreductase 1 (NQO1), glutathione *S*-transferase (GST), cytochrome P450, γ-glutamylcysteine ligase catalytic (GCLC) and modifier subunit (GCLM), superoxide dismutases 1-3 (SOD1-3), glutathione reductase (GSR), thioredoxin reductase (TRxR), thioredoxin (TXN), catalase (CAT), glutathione peroxidase (GPx), and non-enzymatic antioxidants like glutathione, thioredoxin and ferritin [63,71]. Plasma-generated reactive species were similarly translated via the redox-sensitive Nrf2 signaling, leading to an activation of Nrf2-ARE targets, in particular GPx, CAT, SODs, HMOX1, NQO1 and enhanced antioxidant defense [42]. In an in vivo wound model, a more robust and accelerated physiological response was obtained by activating of Nrf2 [33]. ROS production was profoundly elevated in HMOX1 knockout mice [79], showing that HMOX1 is able to metabolize high levels of reactive intermediates. Furthermore, a pharmacological activation of Nrf2 or a knockdown of Keap1 supports wound closure in diabetic mice [80,81].

### 2.3. Plasma-Induced Angiogenesis and Immunomodulation

In addition to its cyto-protective effects, an increasing number of studies support the pivotal role of Nrf2 in angiogenesis [82,83]. The activation of Nrf2 reduces oxidative stress in endothelial cells and suppresses inflammatory responses that may lead to several diseases [84]. Upregulation of Nrf2 by a chemical activator prevents diabetes-induced Erk activation and insulin-signaling downregulation in diabetic patients [85]. Although Nrf2 may promote vascular development via protection of retina from hyperoxia-induced oxidative stress [86], Nrf2 dysfunction may be a potential mechanism underlying impaired angiogenesis and microvascular rarefaction in aging [87]. Plasma-induced regulation of Nrf2 and down-stream targets coincides with the physiological features of wound healing, such as re-epithelialization [35,88], angiogenesis [33], oxidation of lipid layer, and TGFβ signaling [89]. TGFβ signaling is crucially involved in inflammation, angiogenesis, granulation for tissue formation and overall epidermal maintenance [90]. The beneficial activity of plasma is promoted by the production of a plasma-specific signature of inflammatory, growth and angiogenetic factors (e.g., fibroblast growth factor, FGF; vascular endothelial growth factor, VEGF; keratinocyte growth factor, KGF; cyclooxygenase 2,COX2; cluster of differentiation 31, CD31; protein kinase B; Akt etc.) in vitro [39] and in vivo. Regeneration of full-thickness wounds occurred after an early infiltration of neutrophils and macrophages concomitant with a balanced expression of pro- (e.g., IL-1β, IL-6, TNFα) and anti-inflammatory (TGFβ) mediators [33].

### 2.4. Estimations of Risks after Plasma-Induced Activation of Nrf2 Signaling

Some studies suggest an oncogenic characteristic of Nrf2 causing a constitutive activation during early phases of skin tumorigenesis [91], resistance to chemotherapy [92], and an induction of a cancer-associated fibroblast phenotype [93]. Although small amounts of constitutively nuclear localized Nrf2 maintain cellular redox homeostasis through regulation of basal expression of antioxidant genes, after plasma treatment, a transient or persistent activation of Nrf2 does not further increase the cytoplasmic-to-nuclear translocation, thereby preventing cancer formation [42]. Moreover, our data highlight the pivotal role played by Nrf2 in regulating cellular adaptions in response to a repeated cold plasma-induced redox change [94]. Risk estimation studies permitted a clinical application of plasma due to the fail of mutagenicity, genotoxicity [95,96,97], excessive inflammation, and tumor formation [88]. As such, anatomical magnetic-resonance imaging, positron emission tomography-computed tomography, histological and immune histochemical analysis as well as quantitative PCR of several tumor markers showed no apparent signs of tumor manifestation in any of the investigated structures and organs, suggesting that cold plasma treatment is tissue compatible and not damaging to the skin per se. Moreover, other pathways (e.g., Nrf1/3) can partially compensate functions of Nrf2 as shown by Nrf2 knockdown experiments in plasma-treated epidermal keratinocytes (manuscript submitted). Nrf2 in myeloid cells being dispensable for wound healing also indicates the presence of additional antioxidant defense strategies of these cells that compensate for the loss of Nrf2 even in skin wounds [98].

### 2.5. p53 and MAPK Signaling, Directly Influence Nrf2 Activity

The primary event in down-stream signaling of Nrf2 is the recognition of plasma-generated reactive species by specific ROS sensors, e.g., Keap1. Recently, an important new paradigm was introduced by which p53 can mediate a two-phase Nrf2 response; for example, to determine cell fate [99] as well as the cross-talk between oxidative stress (e.g., Nrf2 signaling) and DNA-damage (p53 activation) to define the tipping points where cell injury may switch from adaptation to injury [100]. The nuclear transcription factor and tumor suppressor protein p53 is a key coordinator of oxidative stress [101]. p53 acts as an antioxidant at low levels of oxidative stress to ensure cell survival as well as cellular protection mechanisms and, conversely, p53 has pro-oxidative activities to further increase stress levels to trigger cell death, e.g., apoptosis [102]. Moreover, the redox balance influences the maintenance of cell proliferation rhythms like the cell cycle [103]. p53 signaling can suppress the Nrf2-dependent transcription of antioxidant responsive genes to promote cell survival, apoptosis or cell cycle arrest in a p21-dependent manner [104]. These findings are consistent with the early cell proliferation supporting effect required for rapid tissue repair observed after a transient inhibition of p53 [105], which was also found in a dermal full-thickness wound model upon plasma treatment (manuscript submitted). Several downstream targets of p53 participate in regulation of cellular ROS levels [106], which further influence the activation of all three major mitogen-activated protein (MAP) kinases: p38, the extracellular signal-regulated kinase (Erk), and the stress-activated c-Jun N-terminal kinase (Jnk) [107]. These molecules are also central in cellular Nrf2 signaling processes such as proliferation, differentiation, general stress response [108,109,110,111], and even influencing cell migration [112,113]. The pattern of regulated MAPK expression in different cell settings has also been described after plasma treatment [114,115,116,117]. Moreover, Jnk and p38 modify Nrf2 and p53 activity by phosphorylating it [118,119,120,121] and thus establishing a cross-talk between Nrf2, p53 and MAP kinase signaling [122,123,124]. Altogether, targeting Nrf2 through plasma may offer a novel and better alternative for the therapeutic management of wounds in diabetic patients.

### 2.6. Junctional Proteins in Plasma-Assisted Wound Healing

Interestingly, the gap junction protein connexin 43 (Cx43) might enhance the activation of Nrf2-ARE pathway by means of inhibiting tyrosine kinase c-Src activity to hinder the nuclear export of Nrf2, ultimately attenuating renal fibrosis in diabetes [125]. Cx43, a gap junctional complex component, is a target of interest in dermal wound healing, since it has essential roles in homeostasis and disease [126]. Inhibition of Cx43 by transient blocking of channels with Cx43 mimetic peptides was associated with a significant decrease of ulcer areas, showing that wound healing in diabetic wounds is enhanced when Cx43 levels are reduced [127,128,129]. After plasma treatment, a significant reduction of Cx43 expression was observed in epithelial cells, suggesting a wound closure promoting feature of plasma, especially in chronic and slowly-healing wounds [35]. Indeed, in deep skin layers, Cx43 was transiently upregulated in fibroblasts in the first hours after wounding, as well as during granulation tissue formation and maturity, indicating a localized modulation of Cx43 levels [129].

However, elevated levels of Cx43 had a negative effect on the migratory ability of skin cells [130]; they inhibit the ability of fibroblasts and keratinocytes to migrate into the wound bed and to heal wounds [131]. Skin cells such as keratinocytes and fibroblasts appear to play an important role in migration, differentiation, and re-epithelialization in the final stage of wound closure. After single treatment, plasma had a modulating effect by facilitating cell progression as well as cell motility [33,34,35,42]. For acute redox stress, plasma notably repressed cell migration, which is in agreement with previous results using a carcinoma cell line [132]. Interestingly, cell migration activity partly recovered afterwards, pointing to an adaption of keratinocytes to periodic redox challenges [94]. Moreover, in wound healing, cell migration through connective tissue requires adhesive cell-matrix interactions and ECM contraction, and is mediated by surface integrins and other adhesion molecules [133]. One of the main tasks of fibroblasts is the synthesis of new granulation tissue to recover connective tissue and components of the extracellular matrix [134], which could be demonstrated in plasma-treated fibroblasts, particularly at early stages [33,135]. Overall downregulation of several integrins [35] offers another explanation of the observed changes in cell morphology, including increased motility, rearrangement of actin filaments or stabilization of cytoskeletal architecture. Additionally, the findings support the assumption that certain aspects of Cx43 function could be used for beneficial strategies in wound management and that epidermal as well as dermal skin cells may play a central role in plasma-mediated wound healing.

## 3. Clinical Observations with Cold Physical Plasmas in Dermatology

Although a huge number of plasma sources for biomedical applications are described, only three plasma devices are CE-certified medical plasma devices class II. This includes the argon-driven plasma jet kINPenMed (neoplas tools GmbH, Greifswald, Germany), the microwave-driven Adtec SteriPlas (Adtec Plasma Technology, Adtec Europe, Hunslow, UK), and the dielectric barrier discharge (DBD)-based PlasmaDerm^®^ (CYNOGY GmbH, Duderstadt, Germany). All three plasma devices are based on comprehensive physical, molecular biological, pre-clinical and clinical characterization [33,43,47,136,137]. Generally, standardization of devices is in the focus of the plasma community to compare and identify key process parameters, plasma components, and experimental conditions. First success in translating plasma source protocols and biological application culminated in an unique standardization protocol with DIN-specification 91,315 “General requirements for medical plasma sources”, which was based on the characterization of the plasma jet kINPen MED [53].

In clinical dermatology, cold plasmas are mainly used for the treatment of chronic wounds and pathogen-based skin diseases, in which stimulation of tissue repair and decontamination through killing of microorganisms are combined [38,52,138]. Using DBD, a clinical trial was performed to investigate plasma treatment of chronic wounds [139]. A prospective clinical trial was also conducted using the MicroPlasSter to determine the bacterial load reduction on wounds [62,140], and to show beneficial effects of plasma in patients with infected skin blisters [141]. Similarly, pilot studies or case reports were undertaken using kINPen-generated plasma, showing a decrease of bacterial load [142], a skin recovery after laser-induced skin lesion [143,144,145], a wound healing of ulcer [146,147], as well as a beneficial treatment of psoriasis vulgaris [148].

Nevertheless, the linkage between molecular characterization and clinical protocols will remain a key challenge in plasma medical research in dermatology in order to push healing of chronic wounds in the right direction. To understand mechanisms of wound healing and to deliberate about whether plasma treatment is beneficial, major aims include (i) the modulation of plasma components by tuning of feed gas [149,150,151,152]; (ii) the construction of application-oriented plasma sources, e.g., DBD, jet or microwave devices; (iii) the clinical classification into responder or non-responder; and (iv) the identification of potential biological markers for plasma therapy (currently reviewed in [43]). To answer a broad spectrum of open questions, the prominent aim of plasma medical research consists in the creation of molecular fingerprints of a plasma-triggered wound response.

## 4. Summary and Highlights in Plasma-Induced Wound Healing

**Figure 3:**
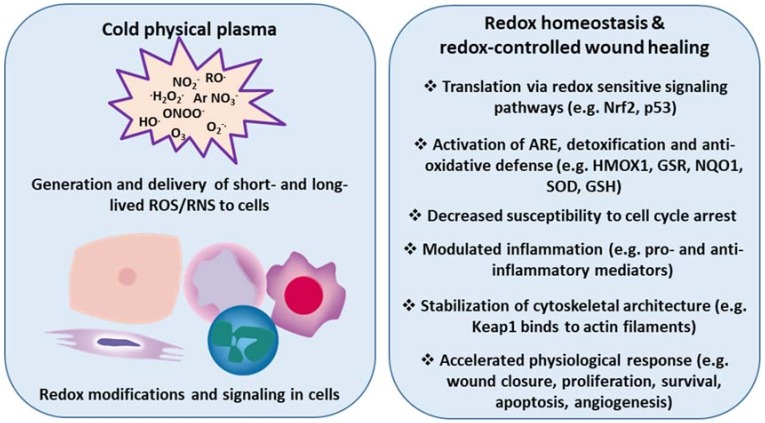
Cold physical plasma-derived generation and delivery of ROS/RNS triggers redox modifications and accelerates physiological responses in wound healing. Redox sensitive signaling pathways (e.g., Nrf2, p53) provide protection from excess ROS/RNS to eventually re-establish redox homeostasis. Cold physical plasma-derived ROS/RNS are efficient triggers of such signaling events in skin cells in vitro and in vivo. Accordingly, plasma treatment promotes several cellular and tissue responses linked to molecular signatures accompanying enhanced wound healing.

Based on findings, we and others have hypothesized that manipulating the redox state of wounds accelerates wound healing by tuning inflammation via redox signaling, regulation of leukocyte traffic and phenotype, and chemokine/cytokine patterns, ultimately fostering skin cell migration into the wound bed. Given its potential (e.g., to kill microorganisms, stimulate cell proliferation, promote tissue regeneration, and modulate inflammation in tissues), cold physical plasma appears to be a promising biomedical tool for the treatment of chronic wounds. Here, we describe that redox-mediated pathways, particularly Nrf2 signaling, are key modulators, not only in wound healing, but also in their promotion via cold plasma treatment (Figure 3). 

Key events are:

- Chronic wounds display subacute inflammation with cellular senescence and bacterial burden
- Plasma accelerates wound healing by different mechanisms of action during the consecutive phases of wound healing
- Plasma controls Nrf2 signaling and inflammatory response in skin cells
- Plasma stabilizes the scaffolding function and actin network in dermal fibroblasts
- Plasma induces changes in Cx43 expression, which could be used for beneficial therapies
- Plasma as a therapeutic option of mild pro-oxidant therapy in chronic (diabetic) wound healing

## Abbreviations

ARE	antioxidant response element
Keap1	Kelch-like ECH-associated protein 1
Nrf2	nuclear factor-erythroid 2-related factor 2
ROS	reactive oxygen species
RNS	reactive nitrogen species
